# Supplementary material for: Variability in tactical behavior of futsal teams from different categories
Source: PLoS One. 2020 Mar 17;15(3):e0230513. doi: 10.1371/journal.pone.0230513 (PMC7077877; doi:10.1371/journal.pone.0230513)
Supplement: Attachment — Submitted filename: Response to the Reviewers.docx [file pone.0230513.s002.docx]

**Responses to the reviewers**

After incorporating the recommendations of the Academic Editor, we are resubmitting the paper **“Variability in tactical behavior of futsal teams from different categories”** for evaluation. We have addressed all the points raised in the review and are thankful for the editor and reviewers’ suggestions, which contributed positively to the quality of the study.

We tracked all the changes in our manuscript and have added point-by-point responses to the reviewers’ comments as follows:

**Reviewer #2:**

a) **All comments of the Academic Editor and Reviewers have been addressed. Therefore, I accept the manuscript in the current form.**

Thank you for the reviewer’s suggestions, which contributed positively to the quality of the study.

**Reviewer #3:**

a) **The article “Variability in tactical behavior of futsal teams from different categories” has a very relevant theme and I consider it to be appropriate to the scope of the Plos One. The variables of tactical behaviors in Futsal need further studies.**

We appreciate the reviewer's comments and suggestions. We have addressed all the points raised by the reviewer, as follows, in point-by-point responses.

b) **A suggestion for better clarity of the work and possible discussions about the athletes' tactical behaviors, would it be to put more information about the registered games, for example, were they games of the qualifying or final phase championship? Was it necessary to win to continue in the championship? We have observed that the tactical behavior of some athletes changes according to the difficulty or value of the game played. I think it would be interesting to make this information about games clear at work, even for new questions in the future.**

Thank you for this suggestion. We added detailed information to the corresponding paragraph.

We would like to thank the reviewers and editor for their contributions and suggestions, which contributed positively to the quality of the study. We hope that the revision of our manuscript meets the publication standards of PLOS ONE.

The authors.
